# Dietary effects of lutein-fortified chlorella on milk components of Holstein cows

**DOI:** 10.1186/s40064-016-2622-6

**Published:** 2016-06-28

**Authors:** Jin-Young Jeon, Keun-Kyu Park, Kyung-Woo Lee, Seung-Wan Jang, Byung-Hern Moon, Byoung-Ki An

**Affiliations:** Department of Animal Science and Technology, Konkuk University, Seoul, 05029 Republic of Korea; Department of Health Food Research and Development of Daesang Corp, Icheon, 17384 Republic of Korea; Celltech, Co., Ltd., Eumseong-gun, Chungbuk 27622 Republic of Korea

**Keywords:** Chlorella, Milk yield, Milk composition, Lutein, Dairy cows

## Abstract

This study was conducted to investigate the dietary effect of conventional or lutein-fortified chlorella on milk production and lutein incorporation in milk. Fifteen Holstein cows in mid-lactation were used in a 3 × 3 Latin square design each with a 21-day period. Cows were top-dressed daily with 30 g of conventional or lutein-fortified chlorella for 3 weeks. Cows without chlorella served as the control. The feed intake and milk yield were not affected by dietary treatments. The concentrations of milk protein and solids non-fat in groups fed diets containing both conventional and lutein-fortified chlorella were significantly higher than those of the control group (*P* < 0.001). There was no significant difference in content of milk fat among groups. The levels of plasma glutamic oxaloacetic transaminase, glutamic pyruvic transaminase, interferon-gamma and interleukin-2 were not influenced by the dietary treatments. Lutein content in milk was significantly increased in groups fed lutein-fortified chlorella as compared with those of conventional chlorella and control, respectively (*P* < 0.01). These results imply that conventional and lutein-fortified chlorella has positive effects on milk components and the use of lutein-fortified chlorella in a dairy diet is effective in the production of milk enriched with lutein.

## Background

Lutein is a natural fat soluble xanthophyll carotenoid occurring as a pigment in some plants and algae. Food sources of natural lutein include green and yellow vegetables and fruits, egg yolks and breast milk (Sommerburg et al. 1998). Several carotenoids, such as lutein and zeaxanthin, occur in significant concentrations in human macula and retina and they are often referred to as macular pigments (Sommerburg et al. 1998). Particularly, lutein, has been known to reduce aged-macular degeneration and cataracts (Olmedilla et al. 2003) and has a strong antioxidant capacity (Sindhu et al. 2010).

Chlorella, a genus of unicellular green algae, is a good source of lutein. Compared with higher plants, chlorella has an advantage of being able to be cultivated in bioreactors on a large scale as a continuous and reliable source of product (Jeon et al. 2012). In previous studies, we found that the lutein content of the chicken eggs was greatly increased after feeding of chlorella powder to commercial laying hens (Jeon et al. 2012). Recently, there have been increasing interests in dietary supplementation of carotenoids and vitamin E to enhance the composition of the fat soluble fractions, particularly lutein, in milk and dairy products (Calderon et al. 2007). In addition, lutein in its role as an antioxidant directly influences nutritional quality of the dairy products (Antone et al. 2012). Therefore, we evaluated the dietary effects of conventional chlorella and lutein-fortified chlorella on milk components and lutein content in milk of dairy cows.

## Methods

### Animals, diets and management

The conventional and lutein-fortified chlorella powders were provided by Daesang Corp. Lutein-fortified chlorella was produced by modifying some elements, such as EDTA-2 Na and trace metal solutions, included in the heterotrophic culture media as described by Jeon et al. (2014). Samples were first analyzed for moisture (Method 934.01), crude protein (Method 976.05), ether extract (Method 920.39), and crude fiber (Method 978.10) using AOAC International (1995). Lutein contents of both chlorella were determined according to the method of Schlatterer and Breithaupt (2006). Proximal composition and lutein contents of conventional and lutein-fortified chlorella are shown in Table 1.

**Table 1:** Proximal composition and lutein concentration of conventional and lutein-fortified chlorella

Analytical items	Conventional chlorella	Lutein-fortified chlorella
Moisture (%)	5.4	3.4
Crude protein (%)	60.6	61.2
Ether extract (%)	12.8	7.6
Crude fiber (%)	13.0	12.0
Crude ash (%)	4.5	5.2
Lutein (mg/g)	4.0	9.1

On a farm, fifteen cows with mid lactation (mean body weight, 628 ± 20 kg; 150–180 days of lactation) were selected from seventy cows based on parity, lactation period, and milk yield. The cows were randomly assigned to three dietary groups: control without chlorella and two test groups fed diets with conventional or lutein-fortified chlorella. They were used in a 3 × 3 Latin square design each with a 21-d period. All cows received the same commercial diet (1620 kcal/kg of NE$\ell$; 15.5 % crude protein). Cows were provided with 15 g (30 g/d) of conventional or lutein-fortified chlorella top-dressed at the feeding time during 3 wks. During the whole experimental period, the cows were housed in a 9 × 10 m free stall barn with no restriction of water consumption. Individual feed intake was measured by locking each feeding gate during the feeding time. Diets were formulated to meet or exceed the nutrient requirements of a 500 kg lactating cow according to NRC (2001) as shown in Table 2. Diets were provided twice daily as TMR ratio of forage to concentrate of 35:65. The feed intake was recorded every day. All animal care procedures were approved by Institutional Animal Care and Use Committee of Konkuk University.

**Table 2:** Formula and chemical composition of experimental diet

Ingredients	%
Corn	37.00
Lupin, flaked	3.60
Wheat	8.30
Rye	5.00
Wheat bran	6.00
Corn gluten feed	7.00
Soybean meal	13.30
Cotton seed meal	8.00
Rapeseed meal	4.00
Molasses	5.00
Limestone	1.60
Salt	0.40
Tricalcium phosphate	0.40
Vitamin and mineral mix^a^	0.40
Sub total	100.00
Calculated values^b^ (%)	
Dry matter	86.90
Crude protein	17.25
Ether extract	2.70
Crude fiber	5.80
NDF	15.92
ADF	7.34
Ca	0.91
Available P	0.65
Total digestible nutrients (%)	70.85

^a^Vitamin and mineral mixture contained the following nutrients per kg of diet: vitamin A, 6000 IU; vitamin D, 2000 IU; vitamin E, 30 IU; Fe, 70 mg; Mn, 95 mg; Zn, 85 mg; Cu, 6.5 mg; I, 1.0 mg

^b^The values were calculated on the basis of Standard Tables of Feed Composition in Korea (2012)

### Sampling and measurements

Cows were milked two times daily in their stall and milk yield was recorded every day. Milk samples were collected on weekly basis and concentrations of fat, protein, solids non-fat (SNF) and lactose were analyzed using a Fossomatic-605 infrared analyzer (Foss Electric, Hillerod, Denmark). An aliquot of milk was prepared separately for further analyses.

Lutein contents in milk were also determined according to the method of Schlatterer and Breithaupt (2006), with some modification. In brief, an aliquot of samples was placed in a round-bottom flask with 45 mL of ternary solvent mixture (light petroleum/ethyl acetate/methanol; 1:1:1, *v/v/v*). Two milliliters of distilled water was added to the flask to facilitate separation. The separation was involved two immiscible liquid phases, the upper layer phase was recovered. After vacuum evaporation (50 mBar, 30 °C, 10 min), the extract including fatty residues was transferred to the volumetric flask with TMBE/methanol (1:1, *v/v*) to a total volume of 10 mL. The extracts were filtered through a No. 6789 0.45 µm filter membrane (Whatman International Ltd., Maidstone, England) and assayed using high-performance liquid chromatography (HPLC; Beckman Coulter Inc., CA, USA).

At the end of each period, blood samples were taken from the jugular vein in heparinized vacutainer tubes. The heparinized tubes were immediately chilled on ice box after sampling, centrifuged at 2000×*g* for 15 min and stored at −60 °C until analysis. The levels of glutamic oxaloacetic transaminase (GOT), glutamic pyruvic transaminase (GPT) and total cholesterol (TC) were measured according to the colorimetric method using a modular system biochemical analyzer (Hitachi Ltd., Tokyo, Japan). Bovine interferon-gamma (IFN-γ) was measured using the Bovine IFN-γ ELISA Kit (Cusabio Biotech Co. Ltd., Wuhan, China) as suggested by the manufacturer. The concentration of plasma interleukin-2 (IL-2) was also measured using the Bovine IL-2 ELISA Kit (Cusabio Biotech Co. Ltd.). The samples and standard curves were measured in triplicate.

### Statistical analysis

Data were analyzed as a 3 × 3 Latin square design using PROC MIXED of SAS (SAS 2002). Dietary treatments were considered fixed whereas cows and periods were random variables. The model was represented as follows:

$${\text{Yijk}} = \upmu + {\text{Ti}} + {\text{Pj}} + {\text{Ck}} + {\text{Eijk}}$$

where Yijk is the observation, µ is the overall mean, Ti is the effect of treatment or diet, Pj is the effect of period, Ck is the effect of cow and Eijk is the random error. Milk yield and milk composition data from the covariance period were used as covariates for their respective variables. Significant differences in observed means were determined using multiple range test at the level of *P* < 0.05.

## Results and discussion

The dietary effects of conventional or lutein-fortified chlorella on lactating performance are shown in Table 3. Feed intake was not influenced by the dietary treatments and both chlorella were completely consumed by the experimental animals. There was no significant difference in milk yield and content of milk fat among groups. The concentration of milk protein and SNF in groups fed chlorella were significantly higher than those of the control group (*P* < 0.001), although there was no difference in lactose content. Somatic cell count (SCC) was not affected by dietary treatments.

**Table 3:** Dietary effects of conventional or lutein fortified chlorella powder on yield and components of milks in dairy cows

Items	Control	T1	T2	Pooled SEM	*P* value
Feed intake (kg/head/day) (on DM basis)	22.8	23.1	23.0	0.58	0.630
Milk yield (kg/head/day)	29.6	30.6	29.6	0.91	0.600
Milk components (g/kg)					
Fat	48.9	49.8	53.3	1.70	0.170
Protein	32.9^c^	34.6^b^	36.3^a^	0.60	0.001
Lactose	45.3	46.3	46.3	0.40	0.100
SNF	82.8^b^	85.4^a^	87.3^a^	0.70	0.001
SCC (cell/mL)	207,666	65,656	68,239	63,985	0.200

Control, basal diet; T1, basal diet + conventional chlorella powder; T2, basal diet + lutein-fortified chlorella Powder

*SNF* solid non-fat, *SCC* somatic cell count

^a–c^ Mean values with different superscripts within the same row differ significantly (*P* < 0.05)

The positive effect of cows fed chlorella on milk protein and SNF may be mediated through the lutein derived from chlorella and/or changes in ruminal fermentation. Xu et al. (2014) reported that supplementation of lutein in the diet can improve milk components, such as fat and lactose, and reduce SCC in lactating cows. The ruminal fermentation by microbes enables growing ruminants to utilize cellulose from chlorella very efficiently (Chowdhury et al. 1995). Feeding algae, including chlorella and *Scenedesmus*, increases fiber digestibility and feed efficiency in growing calves (Chowdhury et al. 1995). Milk yield and/or composition can be greatly influenced by changes in ruminal fermentation via microbial activity (Li et al. 2012). But there are little data available concerning the effect of dietary chlorella on ruminal fermentation in dairy cows. The possibility of changed ruminal fermentation should not be precluded and further study is required to clarify dietary effects of chlorella on ruminal micro-organisms. Milk components are economically important to both milk producers and processors and nutritionally important to consumers. The present results suggest that the conventional or lutein-fortified chlorella is effective in improving milk protein and SNF when applied to dairy cow diets.

The dietary effects of conventional or lutein-fortified chlorella on the concentration of blood profiles are shown in Table 4. The levels of GOT, GPT and TC were not affected by dietary treatments. Measurement of blood GOT and GPT activities indicative of liver and tissue damages in animals is a valuable tool to determine a safe application for new feedstuffs and feed additives (Diaz et al. 2003). In a previous study, we found that conventional chlorella powder appeared non-toxic at the inclusion rate of 0.5 % without adverse effects on physiological status in laying hens (Jeon et al. 2012). The lutein-fortified chlorella used in the present study also was safe as a feed additive in dairy cows based on measurement of blood GOT and GPT.

**Table 4:** Dietary effects of conventional or lutein fortified chlorella powder on blood profiles in dairy cows

Items	Control	T1	T2	Pooled SEM	*P* value
GOT (IU/L)	78.6	78.9	74.9	7.25	0.910
GPT (IU/L)	16.4	16.0	15.9	0.76	0.890
Total-C (mg/dL)	233.9	223.6	235.1	7.89	0.490
IFN-γ (µg/mL)	1.04	1.12	1.23	0.17	0.740
IL-2 (µg/mL)	0.12	0.12	0.29	0.09	0.330

Control, basal diet; T1, basal diet + conventional chlorella powder; T2, basal diet + lutein-fortified chlorella Powder

*GOT* glutamic oxaloacetic transaminase, *GPT* glutamic pyruvic transaminase, *Total*-*C* total cholesterol, *IFN*-*γ* interferon-γ, *IL*-*2* interleukin-2

Some studies suggest a variety of physiological and pharmacological effects of chlorella powder and extracts of chlorella, including lipid metabolism, immunomodulatory and antibacterial activity (Hasegawa et al. 1997; Shibata et al. 2001). An et al. (2008) reported significantly increased levels of serum IFN-γ in chlorella fed mice as compared with the non-fed control, especially under the condition of energy-protein malnutrition. In contrast, Queiroz et al. (2002) found that chlorella extract administration did not affect levels of all cytokines measured in normal/non infected mice. In this study, the plasma IFN-γ and IL-2 in groups fed diets with lutein-fortified chlorella were slightly higher than those of other groups, but not significantly (Table 4). Therefore, it is likely that immune-stimulating effect by dietary chlorella does not always occur because differences in animal species, dosage levels, feeding frequency and health status.

The dietary effects of conventional or lutein-fortified chlorella on the concentration of milk lutein are shown in Table 5. The lutein contents of milk in groups fed diets with conventional and lutein-fortified chlorella were significantly increased as compared with control (*P* < 0.01). The content of lutein in milk from cows fed lutein-fortified chlorella was also higher than that of conventional chlorella (*P* < 0.01). The predominant xanthophyll recovered in milk is lutein, accounting for 12–25 % of total carotenoids (Martin et al. 2004; Calderon et al. 2006). However, the content of milk lutein is not enhanced significantly following a shift from a hay diet to diets with increasing levels of carotenoids (Calderon et al. 2007). In this study, the level of milk lutein in the group fed a diet containing lutein-fortified chlorella was significantly increased as compared with those of control and conventional chlorella, respectively (*P* < 0.01). Because little information on the dynamics of lutein in ruminants is available, the interpretation of present results is difficult. However, dietary lutein is readily absorbed into the blood-stream and is taken up by various tissues in humans (Parker 1989) and rodents (Chew et al. 1996). Chlorella intake improves the carotenoid status of breast milk in healthy pregnant women, with breast milk lutein content being increased by 2.6-fold (Nagayama et al. 2014). In our previous study, the lutein contents of serum and growing oocytes in layers fed diet with conventional and lutein-fortified chlorella were significantly increased than that of control without chlorella (An et al. 2014). Therefore, it is assumed that chlorella lutein is absorbed into the blood and is transported to the liver and the mammary gland in dairy cows.

**Table 5:** Dietary effects of conventional or lutein fortified chlorella powder on lutein concentration in dairy cows

Item	Control	T1	T2	Pooled SEM	*P* value
Lutein (µg/L milk)	231.2^c^	494.0^b^	719.4^a^	94.5	0.010

Control, basal diet; T1, basal diet + conventional chlorella powder; T2, basal diet + lutein-fortified chlorella powder

^a–c^ Mean values with different superscripts within the same row differ significantly (*P* < 0.05)

## Conclusions

Dietary chlorella has positive effects on milk components in Holstein cows and the use of conventional or lutein fortified chlorella is effective for the production of lutein fortified milk.
